# Arcuate nucleus glucokinase and dietary glucose intake

**DOI:** 10.18632/oncotarget.5138

**Published:** 2015-08-11

**Authors:** Yue Ma, Steve R. Bloom, James V. Gardiner

**Affiliations:** Section of Investigative Medicine, Division of Diabetes, Endocrinology and Metabolism, Imperial College London, London, United Kingdom

**Keywords:** glucokinase, glucose, carbohydrate

Glucose is the primary fuel for the brain. It has been proposed that the brain senses glucose, and promotes eating behaviours when levels are low. A system regulating glucose intake, driven by hedonic responses generated in the limbic system, has been identified. A taste-independent mechanism within the CNS that promotes glucose intake has been postulated.

Glucokinase is a member of the hexokinase family of enzymes that convert intracellular glucose to glucose-6 phosphate. There are two isoforms: the hepatic form, expressed in the liver, and the neuroendocrine form expressed in the pancreas and the CNS. Both isoforms catalyse the same reaction, but utilise different promoters and are differentially regulated.

The role of glucokinase in regulating glucose homeostasis in the pancreas and liver is well established [1]. Within beta cells, glucokinase acts as part of the glucose sensing system. Glucose enters the cells via the GLUT-2 transporter. It is phosphorylated by glucokinase as part of a metabolic pathway which leads to closure of ATP-sensitive potassium (K_ATP_) channels. This results in cell depolarisation and insulin release. The role of glucokinase in the CNS is less clear. Glucokinase is expressed in multiple hypothalamic regions, including the VMN, PVN & LHA, areas which regulate energy homeostasis and are part of the CNS glucose-sensing system. A role for hypothalamic glucokinase in the regulation of energy homeostasis has been proposed, but never confirmed [2].

We have recently demonstrated that glucokinase activity within the hypothalamic arcuate nucleus is involved in regulation of dietary glucose intake [3]. We found that in fasted rats, glucokinase activity was increased specifically in the arcuate nucleus but not in other regions of the hypothalamus. Increased arcuate nucleus glucokinase activity increased food intake and weight gain in rats. Moreover, it increased glucose intake, but not that of fructose. Increased intake of glucose occurred in preference to intake of other food types. These effects were mimicked by intra-arcuate administration of glibenclamide, a drug which blocks the K_ATP_ channel. Converse effects were obtained by reducing glucokinase activity in the arcuate nucleus and with intra-arcuate injection of diazoxide, a K_ATP_ channel activator. We also identified a likely mechanism involving P/Q voltage-gated calcium channels, NPY release, and action via neuropeptide Y receptors.

Our results suggest that arcuate nucleus glucokinase is acting as part of a glucose-sensing system, analogous to that in the β cell, as part of a central macronutrient regulatory system. A requirement for glucose, rather than calories alone, partly drives food intake and arcuate nucleus glucokinase can regulate this specific intake of glucose. In the absence of a source of pure glucose, the increased glucokinase activity in the arcuate nucleus drives the intake of mixed nutrient foods.

This hypothalamic system may form part of the post-ingestive reward pathway of glucose, or form a separate homeostatic regulatory system regulating glucose appetite, analogous to that described recently in invertebrates [4]. A role in taste-driven intake is less likely, as this is thought to be more responsive to fructose and sweeteners, which do not appear to activate this glucokinase-based glucose-sensing system.

The findings of this study fit with those of other studies. Hypothalamic glucokinase mRNA expression is increased in diet-induced obese and obesity-prone rats [5]. Patients with mutations that decrease glucokinase activity have reduced body weight [6]. Conversely, patients who have mutations associated with increased activity of the neuroendocrine form of glucokinase have increased body weight [7]. Evidence supports the existence of this pathway in humans. This mechanism may explain the observation that diets high in carbohydrate are associated with weight gain in mice and why low glycemic index diets produce weight loss. It also provides a possible CNS mechanism to explain the often-described phenomena of the “sweet tooth” and carbohydrate craving, particularly for high glycemic index foods.
